# Let-7f: A New Potential Circulating Biomarker Identified by miRNA Profiling of Cells Isolated from Human Abdominal Aortic Aneurysm

**DOI:** 10.3390/ijms20215499

**Published:** 2019-11-05

**Authors:** Rafaelle Spear, Ludovic Boytard, Renaud Blervaque, Maggy Chwastyniak, David Hot, Jonathan Vanhoutte, Nicolas Lamblin, Philippe Amouyel, Florence Pinet

**Affiliations:** 1 Inserm, Université de Lille, CHU Lille, Faculté de Médecine de Lille, Institut Pasteur de Lille, FHU REMOD-VHF, U1167-RID-AGE, F-59000 Lille, France; rafaelle.spear@gmail.com (R.S.); nicolas.lamblin@chru-lille.fr (N.L.); philippe.amouyel@pasteur-lille.fr (P.A.); 2 Inserm, Université de Lille, CHU Lille, Institut Pasteur de Lille, FHU REMOD-VHF, U1167-RID-AGE, F-59000 Lille, France; ludovic.boytard@laposte.net (L.B.); maggy.chwastyniak@pasteur-lille.fr (M.C.); 3 Univ. Lille, CNRS, Inserm, CHU Lille, Institut Pasteur de Lille, U1019-UMR8204-CIIL—Center for Infection and Immunity of Lille, F-59000 Lille, France; renaudblervaque@hotmail.com (R.B.); david.hot@pasteur-lille.fr (D.H.); 4 Univ. Lille, INSERM, CHU Lille, Institut Pasteur de Lille, U1011-EGID, F-59000, Lille, France; jonathan.vanhoutte@pasteur-lille.fr

**Keywords:** abdominal aortic aneurysm, microRNA, macrophages, smooth muscle cells

## Abstract

Abdominal aortic aneurysm (AAA) is a progressive vascular disease responsible for 1–4% of the deaths in elderly men. This study aimed to characterize specific microRNA (miRNA) expression in aneurysmal smooth muscle cells (SMCs) and macrophages in order to identify circulating miRNAs associated with AAA. We screened 850 miRNAs in aneurysmal SMCs, M1 and M2 macrophages, and in control SMCs isolated by micro-dissection from aortic biopsies using microarray analysis. In all, 92 miRNAs were detected and 10 miRNAs were selected for validation by qRT-PCR in isolated cells (*n* = 5), whole control and aneurysmal aorta biopsies (*n* = 13), and plasma from patients (*n* = 24) undergoing AAA (over 50 mm) repair matched to patients (*n* = 18) with peripheral arterial disease (PAD) with atherosclerosis but not AAA. Seven miRNAs were modulated similarly in all aneurysmal cells. The Let-7f was downregulated in aneurysmal cells compared to control SMCs with a significant lower expression in M1 compared to M2 macrophages (0.1 fold, *p* = 0.03), correlated with a significant downregulation in whole aneurysmal aorta compared to control aorta (0.2 fold, *p* = 0.03). Significant levels of circulating let-7f (*p* = 0.048) were found in AAA patients compared to PAD patients with no significant correlation with aortic diameter (*R*^2^ = 0.03). Our study underlines the utility of profiling isolated aneurysmal cells to identify other miRNAs for which the modulation of expression might be masked when the whole aorta is used. The results highlight let-7f as a new potential biomarker for AAA.

## 1. Introduction

Abdominal aortic aneurysm (AAA) is a vascular disease with a prevalence of 5%, which mainly affects men older than 65 years. It is defined by the dilation of the abdominal aorta with a natural asymptomatic evolution towards rupture [1]. Among elderly men, 1–4% of all deaths are due to the fact of AAA [2], and it is the 11th leading cause of death in USA [3]. Abdominal aortic aneurysm biomarkers that can be detected easily in blood before aorta rupture would enable identifying the population at risk and to identify the postoperative course in selected population [4,5].

Abdominal aortic aneurysm is a complex disease with a contribution of different cell types [6] associated with marked changes in the composition of the aortic wall including smooth muscle cells (SMCs) which undergo apoptosis induced by matrix metalloproteases (MMPs). This particular form of cell death, called anoikis [7], decreased the elasticity and rigidity of the aortic wall [8]. Inflammatory cells are also key actors and they are the major source of both MMPs [9] and cathepsins [10] involved in AAA. Macrophages play a key role in inflammation in AAA as antigen-presenting cells [11] and by secreting collagenase and elastase-degrading extracellular matrix [12]. Interestingly, two subtypes of macrophages, M1 and M2, with opposite functions have been described: the M1 subtype has proinflammatory properties and the M2 subtype anti-inflammatory properties [13], distinguished by the presence in the M2 macrophages of mannose receptor (MR), also known as CD206 [14]. Recently, we showed that these macrophages are distributed differentially in AAA [15]. Studying the cells implicated in AAA separately provides an opportunity to identify potential new biomarkers [16]. Laser capture micro-dissection (LCM) makes it possible to isolate cells without modifying their morphology or molecular expression. In previous studies, we used LCM to isolate the two subtypes of macrophages and adventitial tertiary lymphoid organs from AAA samples and compared their protein and microRNA (miRNA) profile, respectively [15,17]. Because AAA development and its subsequent expansion are thought to modify the transcriptome and proteome of SMCs and the two macrophage subtypes known to be involved in this disorder, deciphering their specific targets (RNA, miRNA, and proteins) might help to identify biomarkers.

Small non-coding RNAs, called miRNAs, are a new class of elements involved in transcriptional and post-transcriptional regulation. They have also been shown to be stable as secreted molecules in human fluids, in particular plasma [18]. Specific miRNAs, such as miR-29b and miR-21, have been reported to be associated with AAA in different experimental animal models [19,20]. In two other human microarray-based miRNA expression studies, tissue-and plasma-specific miRNA signatures have been reported for human AAA [21,22]. A recent review highlighted the potential of circulating miRNAs as AAA biomarkers for diagnosis and prediction of AAAA growth [23].

The aim of this study was to profile miRNAs in macrophages and SMCs isolated by LCM from aneurysmal aortas. The differential expression of the selected miRNAs was then evaluated by quantitative RT-PCR in each LCM-isolated cell type as well as in entire aneurysmal and control aorta samples. Finally, the biomarker potential of the miRNAs shown to be differentially expressed in the isolated SMCs and macrophages was then tested in plasma from AAA patients.

## 2. Results

### 2.1. Distribution of SMCs and Macrophages in Human Aneurysmal and Control Aortic Wall Samples

First, we determined the distribution of SMCs and macrophages in human control and aneurysmal aorta samples, while bearing in mind that surgical specimens of human AAA collected from patients undergoing open surgery represent the end stage of the disease.

**Figure 1:**
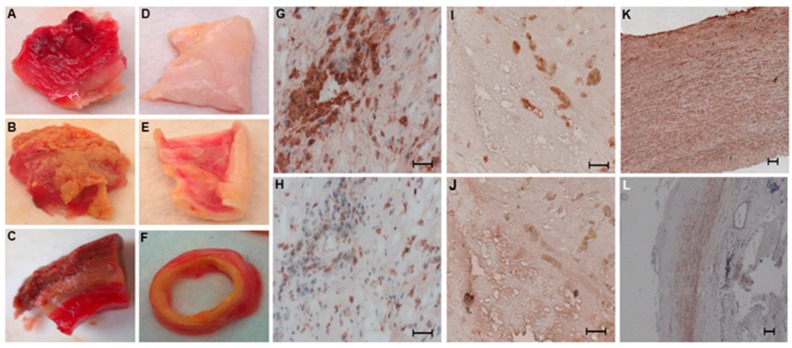
Macroscopic analysis of aneurysmal (**A**–**C**) and control aortas (**D**–**F**) and distribution of M1 and M2 macrophages in aneurysmal aortas (**G**–**J**) and of smooth muscle cells (SMCs) in control (**K**) and aneurysmal (**L**) aortas. Representative internal (**A**,**D**) and external (**B**,**E**) view of aneurysmal and control aortas. Sections of control and aneurysmal aortas were prepared on transversal slides (**C**,**F**) for histology and immunohistochemistry. The entire population of macrophages was stained with an anti-CD68 antibody (**G**,**I**) and the subtype M1 (CD68^+^MR^−^) (**H**) and M2 (CD68^+^MR^+^) (**J**) was discriminated and visualized by an anti-macrophage mannose receptor (MR) antibody (Scale bar: 50 µm). SMCs, stained with α-smooth muscle actin (α-SMA) antibody, were present throughout the control aortas (**K**) and only detected in the media of aneurysmal aortas (**L**) (Scale bar: 100 µm). Immunostaining analysis was performed in every abdominal aortic aneurysm (AAA) sample (*n* = 20) and control aorta sample for SMC (*n* = 14) collected.

Macroscopic analysis of the human aneurysmal and control aorta samples (Figure 1) enabled us to position the luminal versus external side, respectively, for aneurysmal (Figure 1A,B) and control aortas (Figure 1D,E). Biopsy samples were then oriented before dissection into transversal sections (Figure 1C–F). The macrophage subtypes, M1 (CD68^+^MR^−^) (Figure 1G,H) and M2 (CD68^+^MR^+^) (Figure 1I,J), were identified by CD68 antigen staining (Figure 1G–I), which is a pan macrophage marker, and distinguished by the macrophage mannose receptor (Figure 1H–J), only present on the M2 macrophages [14]. As we previously showed, each subtype of macrophage was found in its own distinct area: anti-inflammatory macrophages (CD68^+^MR^+^) in the luminal side of the aneurysmal aortic wall and proinflammatory macrophages (CD68^+^MR^−^) in the more degraded adventitial layer as previously described [15]. No macrophages were detected in control aortas. We then performed α-SMA immunostaining for detecting SMCs: numerous SMCs were present, organized in multilayers in the control human aortic walls (Figure 1K). In contrast, in the aneurysmal aortas, SMCs were only observed in the media (Figure 1L).

### 2.2. Profile of miRNAs in SMCs and Macrophages Isolated by LCM in Human Aneurysmal and Control Aortas

We performed specific immunostaining for the localization of SMCs from the control (*n* = 6) and aneurysmal samples (*n* = 6) and M1 (*n* = 5) and M2 macrophages (*n* = 6) from solely aneurysmal samples. Areas abundant in SMCs and M1 and M2 macrophages were then isolated by LCM. We isolated an average area of 9.8 (4.7–16.2) mm^2^ abundant in the cells of interest corresponding to an average of 784 (314–1508) ng of RNA. The presence of the specific cell types was verified by immunostaining every three 18 µm sections.

**Figure 2:**
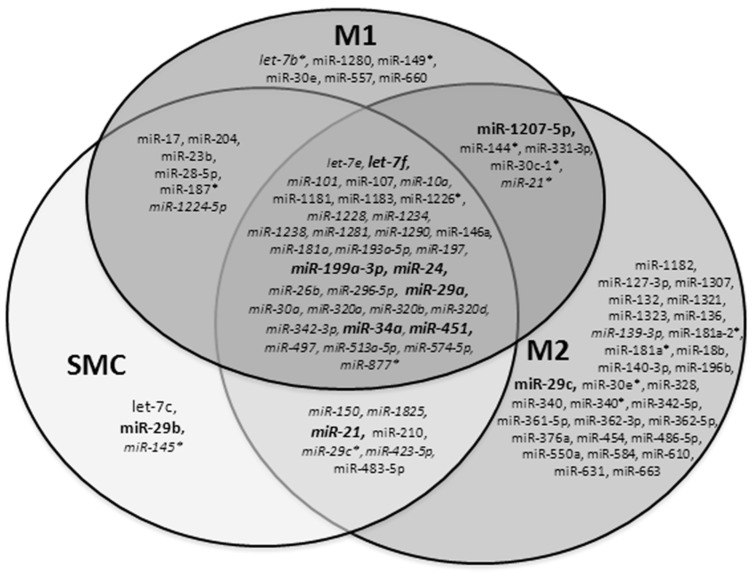
Venn diagram analysis of miRNA screening from laser capture microdissection (LCM)-dissected areas rich in aneurysmal M1 and M2 macrophages and in SMCs isolated from aneurysmal aortas. Isolated cells from two different aorta samples were run in two arrays to screen for 850 human miRNAs: 92 miRNAs were detected in aneurysmal SMCs and M1 and M2 macrophages with 87 miRNAs detected in the macrophages, 38 of them common to M1 and M2 subtypes and 54 in SMCs, and 35 of them were found in both aneurysmal and control cells. MiRNAs detected in control SMCs are indicated in italics. The 10 miRNAs selected for further analysis, using the normalized value of miR-29b as the threshold, are indicated in bold.

Each cell type isolated by LCM from two different biopsy samples was screened, and 92 miRNAs were detected among the 850 human miRNAs screened. In summary, 87 miRNAs were detected in the macrophages with 38 of them in both the M1 and M2 subtypes, and 54 miRNAs were detected in the SMCs with 35 of them in both aneurysmal and control cells (Figure 2). 

We observed that 33 miRNAs were common to all the three types of aneurysmal cells, and 5 were specific of aneurysmal SMCs. Five miRNAs were specific and common to both macrophage subtypes, six specific to M1 macrophages only, and 30 only to M2 macrophages (Figure 2).

Among the 92 miRNAs detected, we set up a threshold for our subsequent selection based on the normalized value of miR-29b in order to ensure correct quantification by quantitative reverse transcription polymerase chain reaction (RT-qPCR). The miR-29b was previously reported to be downregulated in AAA murine models [21]. The following 10 miRNAs were selected for further analysis: let-7f, miR-199a-3p, miR-1207-5p, miR-21, miR-24, miR-29a, miR-29b, miR-29c, miR-34a, and miR-451. Six of them (let-7f, miR-199a-3p, miR-24, miR-29a, miR-34a, and miR-451) were common to both SMCs and macrophages. Both macrophage subtypes contained miR-1207-5p, and both SMCs and M2 macrophages, miR-21. Two miRNAs were specific to a single cell type: miR-29c was only detected in M2 macrophages and miR-29b in aneurysmal SMCs (Figure 2).

### 2.3. Validation of miRNAs Expression in Aneurysmal Cells

**Figure 3:**
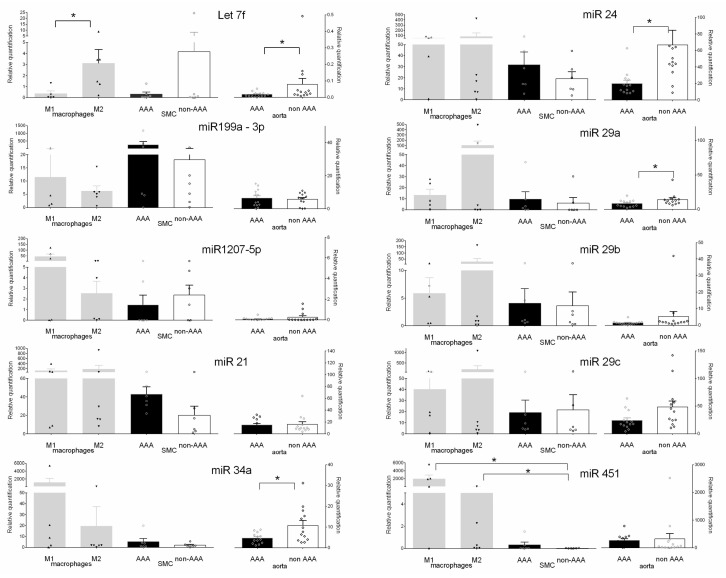
Quantification of the ten miRNAs selected in LCM-isolated cells and in the whole aortic biopsy samples by RT-qPCR with the Δ*C*t method. Differential expression in isolated aneurysmal SMCs (*n* = 6), M1 (*n* = 5), and M2 macrophages (*n* = 6) was quantified with isolated control SMCs as the reference and RNU6-2 for calibration. Differential expression in the whole aneurysmal aorta samples (*n* = 13) was quantified with the control aortas (*n* = 13) as the reference and RNU6-2 for calibration. Data were expressed as mean 2^−Δ*C*t^ ± SEM with individual values indicated by open circles. * *p* < 0.05.

Expression of the 10 selected miRNAs was analyzed by RT-qPCR to validate the miRNA screening performed in aneurysmal cells, i.e., SMCs (*n* = 6), M1 (*n* = 5), and M2 (*n* = 6) macrophages compared to non-aneurysmal SMCs (*n* = 6) (Figure 3). We found that 7 miRNAs were modulated similarly in the three cell types (i.e., SMC/M1/M2) isolated in aneurysmal tissue compared to SMCs isolated from control aortas: let-7f (0.08/0.08/0.75 fold) was downregulated and miR-21 (2.15/5.7/8.9 fold), miR-24 (1.6/2.6/4.1 fold), miR-29a (1.6/2.2/17.3 fold), miR-29b (1.6/2.6/4.2 fold), miR-34a (2.7/563/10 fold), and miR-451 (17/10^5^, *p* = 0.02/434 fold, *p* = 0.03) were upregulated compared to non-aneurysmal SMC (Figure 3). The miR-1207-5p and miR-29c were downregulated in aneurysmal SMCs (0.6/0.9 fold) and upregulated in M1 (18/1.85 fold) and M2 (1.1/8.5 fold) macrophages. Conversely, miR-199-3p was upregulated in SMC (15 fold) and downregulated in M1 (0.6 fold) and M2 (0.3 fold) macrophages (Figure 3). 

To underline the utility of analyzing these cells individually in aneurysmal aorta, we quantified the 10 selected miRNAs in whole biopsy samples from aneurysmal (*n* = 13) and control aortas (*n* = 13) (Figure 3). Only miR-199-3p was slightly upregulated in the whole aneurysmal aorta by comparison to control aortas (1.1 fold). The others were downregulated in aneurysmal aorta with the lower expression for let-7f. Among the 7 miRNAs that were modulated similarly in the three isolated cell types, three were also correlated in the whole aneurysmal aorta with a significant downregulation for let-7f (0.2 fold, *p* = 0.03) in AAA. Three miRNAs were conversely correlated in the whole aneurysmal aorta with a significant downregulation of miR-24 (0.3 fold, *p* = 0.01), miR-34a (0.4 fold, *p* = 0.03) and miR-29a (0.5 fold, *p* = 0.02) (Figure 3).

To evaluate the potential of the 10 miRNAs selected from the microarray as circulating AAA markers, we quantified in plasma their levels in relatively small but discriminating populations (Table 1): peripheral arterial disease (PAD) patients with atherosclerosis but no AAA (PAD-control, *n* = 17) and case patients (*n* = 22) who had both atherosclerosis and AAA (mean aortic diameter: 56.1 mm) [24]. 

**Figure 4:**
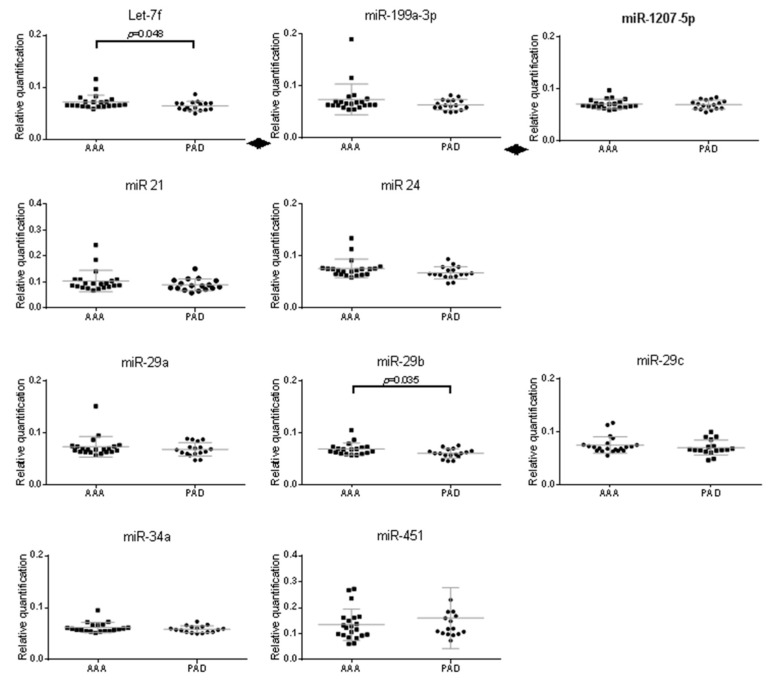
Quantification of the ten miRNAs in plasma from patients with AAA (*n* = 22) and in patients with PAD without AAA (*n* = 17) by RT-qPCR with the Δ*C*t method with Syn-cel-miR-39 for calibration. Data are expressed as the mean Δ*C*t ± SEM and show the individual values obtained for each miRNA quantified. The exact *p*-value is indicated for the significant differential expression between PAD and AAA patients.

Expression of only let-7f (4.7 fold, *p* = 0.048) and miR-29b (5 fold, *p* = 0.035) were statistically significantly upregulated in the plasma of AAA patients compared with PAD patients (Figure 4).

Correlation between their expressions in plasma with AAA diameter was evaluated. Neither the plasma concentrations of let-7f (*R*^2^ = 0.03, *p* = 0.45) and miR-29b (*R*^2^ = 0.67, *p* = 0.42) were significantly correlated with AAA diameter.

## 3. Discussion

Abdominal aortic aneurysm is a complex disease associated with marked changes in the cellular composition of the aortic wall. miRNAs have been implicated in the etiology of a variety of human diseases including cardiovascular diseases [25]. They also play a major role in various biological processes including apoptosis and metabolism [26]. 

Our objective was to analyze cells isolated from human aneurysmal and control aortic biopsies to decipher the miRNAs that may play a role in causing AAA. Until now, miRNA profiling for AAA disease has relied on either experimental AAA murine models [19] or whole aneurysmal aorta biopsy samples [21]. A review has stressed the importance of determining the cellular expression patterns of each miRNA so that downstream studies can be conducted in the appropriate cell type [27]. Abdominal aortic aneurysm and its evolution depend on various mechanisms such as inflammation, apoptosis, and extracellular matrix degradation. The rarefaction of SMCs is secondary to anoikis, that is, their apoptosis induced by their detachment from the extracellular matrix [7]. Inflammatory cells producing cytokines might be responsible for SMC apoptosis [28]. Both M1 and M2 macrophage subtypes are involved in obstructive atherosclerotic arteries as well as in AAA [15,29]. Their distribution in the aneurysmal wall illustrates the pathological balance of inflammation in AAA. The SMCs and macrophages are key cells that may interact in AAA and, therefore, may be a potential source of biomarkers in the blood. 

Laser micro-dissection makes it possible to isolate areas that are abundant in the cells of interest. To avoid contamination of the LCM-isolated areas with a different cell type, only a limited number of samples (two to four for each cell types) were selected for micro-dissection from aneurysmal biopsies and screened in duplicate on the microarray. Together, combining the SMCs and the two macrophage subtypes that we isolated from the control and aneurysmal samples, we detected a total of 92 of the 850 miRNAs for which we screened. Among these 92, microarray profiling has previously found 5 miRNAs (miR-181a-3p, miR-146a, miR-21, miR-331-3p, and miR-204) to be differentially expressed in AAA and control tissues [21]. 

Our finding that 7 miRNAs (let-7f, miR-21, miR-24, miR-29a, miR-29b, miR-34a, and miR-451) among the 10 selected were modulated similarly in all three aneurysmal cell types with only let-7f modulated similarly in the whole aneurysmal aorta biopsies suggests its implication in AAA disease. Interestingly, the miR-29 family has also been described in murine AAA models [19] and is downregulated similarly in aneurysmal compared to control aortas, although it was upregulated in the aneurysmal SMCs and macrophages from LCM-isolated samples. The opposite trends in the expression of the miR-29 family between isolated cells and whole aortas underline the specific role of each cell in the aneurysmal aortic wall. 

We did not detect some miRNAs (miR-516a-5p and miR-1260) previously reported as differentially expressed in AAA; for example, the miRNAs differentially expressed in SMCs from control and AAA explant cultures [30]. This discrepancy may result from a phenotypic change due to the explant culture. Similarly, we did not detect miR-155 which has been shown to be overexpressed in AAA due to the accumulation of T-lymphocytes [31], a cell type not screened in our study. Red blood cells, which are the predominant cells in plasma samples with hemolysis, are reported to be the only cells known to express miR-451 [32]. Accordingly, we found that miR-451 was highly expressed in aneurysmal cells but not in whole aneurysmal aorta, perhaps due to the presence of an intraluminal thrombus.

These data and those of other authors reveal the complexity of aneurysmal walls and show that profiling miRNAs from isolated aneurysmal cells may identify miRNAs for which the modulation of expression might be masked when the whole aorta is used and may serve as sources of potential AAA biomarkers.

Next, we tested the potential of the miRNAs selected as AAA biomarkers by comparing their levels in plasma of the patients with AAA to those with PAD (control group). This control group enables the identification of miRNAs specific to AAA disease, independently of atherosclerosis. Indeed, a study identified 29 circulating miRNAs in patients with AAA compared to control subjects. They validated a significant downregulation of miR-196b which was not maintained by comparison between AAA and PAD cohorts [33]. 

Only two miRNAs were found to be statistically significantly upregulated in plasma from AAA patients: let-7f and miR-29b. The latter and its family members (miR-29a and miR-29c) are considered to be markers of AAA in murine AAA models [25]. Our data reinforce the evidence that miR-29b is a potentially useful circulating biomarker for AAA disease. The significant upregulation of let-7f plasma levels in AAA patients may be related to its higher expression in aneurysmal M2 macrophages compared to M1 macrophages and aneurysmal SMC. Let-7f is reported to be increased in the intima of patients with atherosclerosis obliterans, although no modulation in the serum of these same patients was observed [34]. Interestingly, let-7f has been identified as acting as an inhibitor of inflammatory processes [35]. Here, we compared the plasma levels of let-7f in AAA patients to those in a control population of patients with occlusive atherosclerosis presenting the same cardiovascular risks as previously performed [33]. Our findings suggest that let-7f has strong potential as a biomarker of AAA. The study could be limited by the presence in human population of subjects in which PAD and AAA coexist, since atherosclerosis can be present both in dilated and obstructive forms of large- and medium-caliber arteries [36].

## 4. Materials and Methods 

### 4.1. Human Abdominal Aorta Samples

Our study conformed to the principles outlined in the Declaration of Helsinki. We obtained informed consent in writing from each patient undergoing open surgical repair of an infrarenal aneurysm and recovered the biological samples as surgical waste, in accordance with French laws on medical ethics (CHRU of Lille, France, 02/01/2009). Healthy non-aneurysmal aortas (control samples) came from deceased patients providing multiple organ retrievals, with the written consent of their families and the authorization of the French Biomedicine Agency (PFS 11-004).

Thus, samples of human aneurysmal infrarenal aortic walls were obtained from 20 patients in Haulon’s vascular surgery unit (Hôpital Cardiologique, CHRU Lille, France). Each sample was dissected into transversal slides after orientation of the tissue by macroscopic analysis. Sections were formalin-fixed, paraffin-embedded, and kept at 4 °C or they were snap-frozen in liquid nitrogen, in both cases for further analyses.

Fourteen control abdominal aorta samples to serve as control tissue were collected during multiple organ retrievals, with the collaboration of Pruvot’s transplant unit (CHRU Lille, France). All samples were preserved in normal saline solution at 4 °C.

### 4.2. Human Plasma Samples 

Plasma samples were collected from another AAA population and from control patients with a documented peripheral artery disease (PAD). The AAA and PAD-control populations (Table 1) have been previously described [24]. The LILle Aneurysmal Study (LILAS) was a case-control study that enrolled 42 men with either AAA (*n* = 24) or peripheral artery disease (PAD) (*n* = 18) who required vascular surgery at the same hospital center (Lille, France). Case/AAA patients were eligible if the AAA diameter, measured by abdominal ultrasound, exceeded 50 mm or if it had increased more than 5 mm during the past six months. The PAD patients were eligible if PAD was diagnosed in the aortic, iliac, or femoral arteries and AAA had been ruled out. The hospital’s ethics committee approved both studies, and each patient and subject provided written informed consent.

### 4.3. Histological Analysis and Immunohistochemistry

Immunohistochemical analyses were performed on the 20 paraffin-embedded tissue samples to locate the different cells. We identified macrophages with mouse anti-CD68 (dilution 1/50, EBM11 Clone, ref M0718 DAKO Corporation) antibody for the overall macrophage population and goat anti-CD206 (dilution 1/50, ref SC-34577 Santa Cruz Biotechnologies) antibody to discriminate the CD68^+^MR/CD206^−^ (M1) and CD68^+^MR/CD206^+^ (M2) subtypes. Incubation of primary antibodies lasted for 2 h except for anti-CD206 which lasted for 20 h. The SMCs were identified with mouse anti-alpha-smooth muscle actin (α-SMA) (dilution 1/50, ref M0851 DAKO Corporation) antibody. Immunostaining used the appropriate biotinylated secondary antibodies (dilution 1/200, Vector laboratories), streptavidin-horseradish peroxidase (ABC kit, Vectastin), and the AEC substrate-chromogen system (Sigma) for visualization. Finally, slides were analyzed with an Axioplan 2 microscope, which included an HRc camera (AxioVision-Deconvolution 3D). Negative controls were performed by omission of the primary antibody and substitution with an unrelated primary antibody. As expected, both controls yielded negative results. 

### 4.4. Laser Capture Microdissection (LCM)

Frozen sections (8 μm) of both aneurysmal and non-aneurysmal samples were stained with oil-red O until cellular zones were detected. The adjacent sections were then stained for α-SMA to identify the areas with abundant SMCs and for CD68 and MR to identify the areas with abundant M1 (CD68^+^MR^−^) and M2 (CD68^+^MR^+^) macrophages. Adjacent 18 μm unstained frozen sections were prepared for LCM on PEN membrane glass slides (MDS Analytical Technologies) by dehydration in alcohol and then clearance with xylene (Sigma–Aldrich). The LCM was performed with an ArcturusXT micro-dissection instrument (MDS Analytical Technologies). Micro-dissected areas were collected on CapSure Macro LCM Caps (MDS Analytical Technologies) as previously described [15].

### 4.5. Microarray Analysis

The RNA was extracted from LCM-dissected areas rich in M1 macrophages, M2 macrophages, and SMCs. Each cell type was obtained from two different aortic biopsy samples and was screened in duplicate for miRNA expression with the human miRNA 8 × 15 k microarray (850 known human miRNAs, according to the Sanger database version 12.0, Agilent Technologies). Data are available at the Gene Expression Omnibus (GEO) (www.ncbi.nlm.nih.gov/geo) under the accession number GSE62179. 

### 4.6. miRNA Quantification by q-RT-PCR

Using the miScript II RT kit (Qiagen) with HiSpec Buffer (Qiagen) to focus on mature miRNAs with oligo-dT primers, we reverse-transcribed 100 ng of total RNA. Next, the cDNA was amplified with miScript PreAMP PCR kit (Qiagen). The PCR was then performed with the miScript SYBR Green PCR kit (Qiagen) on a Mx3000P Q-PCR system (Agilent Technologies), according to the manufacturer’s instructions. The PCR miScript Primer Assays (Qiagen) of let-7f (MIMAT0000067), miR-199a-3p (MIMAT0000232), miR-1207-5p (MIMAT0005871), miR-21 (MIMAT0000076), miR-24 (MIMAT0000080), miR-29a (MIMAT0000086), miR-29b (MIMAT0000100), miR-29c (MIMAT0000681), miR-34a (MIMAT0000255), miR-451 (MIMAT0001631), and RNU6-2 were used for q-RT-PCR.

### 4.7. Statistical Analysis

Statistical analyses were conducted using the Mann–Whitney test for differences in miRNA quantification and the Shapiro–Wilk test for correlation between miRNA expression and aortic diameter using GraphPad Prism software. A *p*-value < 0.05 was considered statistically significant.

## 5. Conclusions

In conclusion, our results relied on the utility of analyzing specific cells rather than whole biopsy samples in AAA disease as previously reported [16]. While the samples studied were limited in number, the miRNA profiling of isolated aneurysmal SMCs and M1 and M2 macrophages enabled us to detect miRNA specifically expressed in SMCs and macrophage subtypes. Our data highlighted the complexity of cells and their function in the aneurysmal wall and the potential role of intra- and extra-cellular miRNA regulation. Mechanistic analyses on vascular cells implicated in an AAA wall will help in understanding the specific role of miRNAs in the evolution of the disease. Two miRNAs were differentially modulated in the plasma of patients with AAA, let-7f, and miR-29b suggesting their potential as biomarkers of AAA. Circulating levels of miRNAs can be used as biomarkers of therapeutic success as reported recently after AAA endovascular repair [37]. Further clinical studies will be required to determine if they can be used as diagnostic or prognostic biomarkers.

## Figures and Tables

**Table 1:** Baseline characteristics of the study population.

	AAA (*n* = 24)	PAD (*n* = 18)
Age, years	68.0 ± 6.1	62.3 ± 6.6
Body mass index, kg/m^2^	27.4 ± 3.7	26.3 ± 4.1
Aortic diameter	56.1 ± 2.3	
Cardiovascular risk factors, *n* (%)		
Current smoking	4 (17)	4 (22)
Past smoking	17 (71)	13 (72)
Hypercholesterolemia	14 (58)	12 (67)
Hypertension	15 (63)	10 (56)
Diabetes mellitus	4 (17)	5 (28)

AAA: aortic abdominal aneurysm, PAD: peripheral arterial disease.

## Abbreviations

AAA	Abdominal aortic aneurysm
LCM	Laser capture microdissection
miRNA	microRNA
MMP	Metalloprotease
MR	Mannose receptor
SMC	Smooth muscle cell
PAD	Peripheral arterial disease
